# Role and Characteristics of Personal Care Assistants of Frail Older People with Functional Limitations Ageing in Place in Italy

**DOI:** 10.3390/ijerph19073969

**Published:** 2022-03-26

**Authors:** Maria Gabriella Melchiorre, Sabrina Quattrini, Giovanni Lamura, Marco Socci

**Affiliations:** Centre for Socio-Economic Research on Ageing, IRCCS INRCA—National Institute of Health and Science on Ageing, 60124 Ancona, Italy; s.quattrini@inrca.it (S.Q.); g.lamura@inrca.it (G.L.); m.socci@inrca.it (M.S.)

**Keywords:** ageing in place, living alone, frail older people, functional limitations, personal care assistant, domestic home help, Italy

## Abstract

When older people become frail with functional limitations, and age alone in place, caring support is fundamental for performing daily living activities. The present study aimed to explore the current role and characteristics of privately hired Personal Care Assistants (PCAs) of older people in Italy, in light of the decreasing care availability of the family and the low provision of public services. In the study “Inclusive ageing in place” (IN-AGE), 120 qualitative interviews were carried out in 2019, involving frail older people living at home in three Italian regions: Lombardy, Marche, and Calabria. A content analysis was conducted, in addition to some simple quantifications of statements. Results showed the support of PCAs in 27 cases, mainly when health issues of seniors were referred. In addition, informal and irregular employment contracts were reported. Moreover, a comparison between PCA and Domestic Home Help (DHH, 44 cases), highlighted how they even more provide very similar functions (i.e., home and personal care). The role of PCA emerged as crucial in Italy, especially in the South. Thus, to boost up home services seems necessary for allowing ageing in place, also by integrating PCAs in formal public Long-Term Care (LTC), and by providing incentive systems for regular hiring.

## 1. Introduction

The worldwide population is often ageing in place, meaning at home. Such a context becomes crucial for frail older people, especially when they live alone and have limited physical/functional limitations [1,2]. Regarding the articulated issue of frailty, the absence of a gold standard for its identification emerges [3,4,5]. According to the World Health Organisation (WHO) frailty is defined as “extreme vulnerability to endogenous and exogenous stressors that exposes an individual to a higher risk of negative health related outcomes” [6] (p. 227), which affect “several domains of human functioning (physical, psychological, and social)” [1] (p. 2). Moreover, the Italian Ministry of Health [7] highlights the need to detect frail people in order to provide tailored interventions for preventing disability. This holistic approach, although comprehensive, is nevertheless complex to manage [8,9], and indeed “most of the available frailty screening tools do not address all domains of functioning” [1] (p. 2). In light of these considerations, our study adheres to a more accessible analysis of frailty, referring to a condition linked to ageing [1], and to living alone with limited functional abilities, due to a decline in “multiple physiological systems” [10]. This implies restrictions on independent living and the need for support [11], with existing social welfare provisions for older people becoming crucial [12].

In Italy, the country where this study is focused on, the over 65s represent 23.5% of the total population as of 1 January 2021 [13]. Moreover, in this country, about 50% of people living alone are over 65 [14], and 44% of this population group have severe limitations in performing the overall activities of daily living [15]. The main Long-Term Care (LTC) support for frail older people in Italy is still represented by the informal help of family members, especially children [16,17], who provide care to 86% of older people living alone [18]. Recent studies [19,20] indicated also that in this country, family caregivers are mainly women (60%) in the “middle age” (45–64 years). However, the caring role of the family is currently decreasing, due to the reduction in both household size and cohabitation of parents and children, and conversely to the increased participation of women (being often usual caregivers), in the labour market [21]. Moreover, public care intervention in Italy is marginal. Indeed, public expenditure in LTC provision for older people is about 20% lower than the European average [22,23], and divided among the National Disability Attendance Allowance (IA, *Indennità di accompagnamento,* 52%), residential care (30%), and just 18% is allocated to home services, e.g., Integrated Home Care (ADI, *Assistenza Domiciliare Integrata*) and Home Care Service (SAD, *Servizio di Assistenza Domiciliare*) [24]. More specifically, in 2018 IA (EUR 525,17 per month in 2022) [25] registered 12% of totally dependent users aged 65 and over [19]. Such a scenario, i.e., low supply of public home care and mainly provision of IA, impacts on the use of private support services. According to Albertini and Pavolini [26], the institutional design, coverage and intensity of the public support provision in a country, influence indeed the extent to which households resort to the care market, in particular to purchase home care services for older people [27,28].

In this respect, the main available cash-for-care scheme, i.e., IA, plays a key role in Italy, since it is often used to hire a Personal Care Assistant (PCA), also named “*badante*”, or Migrant Care Worker (MCW) when having a foreign origin [29], both cohabitant/in house and on an hourly basis [30]. In the Italian context, 88% of PCAs are women, and 44% from Est Europe [31]. They are hired especially when care for seniors requires intensive daily care [22,32], and mainly in the Centre-South of the country, whereas a residential care provision prevails in the North [33]. Two thirds of Italian frail seniors living alone have a PCA, and in more than half of cases, the latter work on a daily basis [18]. In particular, among the 75s and over living alone, the use of PCAs is widespread, with 58% relying on them every day, and 18% few times a week [17]. Moreover, in 2019, statistics on regular domestic workers showed 407,000 PCAs (48%) and 441,000 units as Domestic Home Help (DHH) (52%), and thus on a total of about two million of both regular and irregular positions, over half with no formal employment contract were estimated [34]. Some concerns arise also regarding cultural/linguistic issues when PCAs are from abroad, due to possible misunderstandings and different habits between older people and care assistant [17], in addition to costs not affordable for all the families [35].

Despite these criticalities, the employment of PCAs represent, mainly in Mediterranean countries (Italy included), a private solution to manage the challenge of caring an increasing number of frail and dependent older people [36], since these private assistants have largely bridged the gap between the growing demand for care services, the modest LTC’s public service provision, and the reduced care capacity of families [37]. The opportunity for families to privately hire PCAs can allow older people ageing in place, with the possibility to give continuity to social and family relationships [18]. In order to explore the current role of PCAs caring for frail older people with physical limitations living alone in Italy, the paper aims to answer the following research questions: (1) What are the reasons for hiring or not a PCA? (2) What are the main characteristics of PCAs? (e.g., gender, age, nationality, work contract and cost) (3) Are there regional and/or urban/rural differences in this respect? A last/minor question investigated possible differences/similarities between PCAs and DHHs. The analysis of ageing in place, with the support of PCAs, seems relevant in the current context, especially when comparing different territories and in the light of the complex scenario due to the COVID-19 outbreak, where both formal and informal supports for older people seem lacking and inadequate. 

## 2. Materials and Methods

### 2.1. Setting, Sampling and Participants

The paper presents findings from the “Inclusive Ageing in Place” (IN-AGE) study, that involved 120 older men and women aged 65 years and over, who were interviewed in May–December 2019, in urban and rural sites of three Italian regions: Lombardy (North), Marche (Centre), and Calabria (South). These three contexts can in fact well represent the vertical/regional differentiations characterizing this country, with greater socio-economic disadvantage, especially regarding the availability of public services for caring older people, in the South, greater overall development of such services in the North, and with the Centre often showing an intermediate situation in this regard [16]. In particular, the three regions belong to three different welfare/care clusters [33], as follows: mainly cash-for-care, e.g., several beneficiaries of IA, in Calabria; mixed cash-for-care (IA and home care services) in the Marche; and the residential care cluster, with prevalence of beds availability in care facilities, in Lombardy. On the whole, three medium-sized urban cities (Brescia, Ancona, and Reggio Calabria, respectively), and three inner areas [38] (Oltrepò Pavese, Appennino Basso Pesarese e Anconetano, and Area Grecanica, respectively) were included. In each urban area 24 qualitative interviews were carried out (total 72), and 16 in each rural one (total 48). In each region a total of 40 interviews were this collected. The most fragile locations were detected within both urban and rural contexts, by using indicators regarding material, social and territorial vulnerability, e.g., greater presence of older people living alone; share of families living in public housing (*Edilizia Residenziale Pubblica*—ERP); low education level; high unemployment level; and scarce availability of public services [39]. A purposive sample was built [40], with respondents selected as follows [16]: older people living alone at home or with the support of a PCA (cohabitant or on daily/nightly hourly basis, for at least 28 h per week); intermediate mobility (within the home, and outside with the support of a person or aids); absence of cognitive impairment as capacity to answer questions of interview independently; and absence of very close family members who give help (living in the same urban block/rural building). Respondents were recruited with the help of the local sections of Auser (Voluntary association for active ageing), operators of municipal/public home services (e.g., SAD), and further voluntary associations (Anteas, Caritas). These channels were of support for collecting adhesions, names, and contact details (address and telephone numbers) of potential eligible participants, and also for informing them on the purpose of the study. A preliminary verbal consent of seniors to be interviewed, and to communicate personal details to interviewers, was thus obtained [40].

### 2.2. Data Collection and Measures

Qualitative face-to-face interviews were administered by six expert and ad hoc trained researchers (two for each region), with a background mainly as psychologists and sociologists. A semi-structured interview/topic guide was used, with questions exploring the following topics: socio-demographic; family and housing; health status and use of services; daily living activities and functional limitations; care arrangements including PCAs; economic situation of respondents; social isolation and perceived loneliness. In order to explore the topics, several questions were drawn and adapted from previous studies (e.g., [41]). Moreover, validated scales were used to detect the limitations in performing (in autonomy, with help, not able) both Basic and Instrumental Activities of Daily Living (ADLs and IADLs) [42], in addition to questions on two sensory limitations (difficulty in seeing and hearing), and two mobility limitations (going up/down the stairs, and bending to pick up an object) [43,44]. A written informed consent was signed by each participant, and the approval of the Ethics Committee of Polytechnic of Milan was obtained (POLIMI, Research Service, Educational Innovation Support Services Area, authorization n. 5/2019, 14 March 2019). Reassurance was given to participants with regard to confidentiality/privacy and anonymity of the information collected, also following ethical issues according to the European General Data Protection Regulation (GDPR) n. 679 of 27 April 2016 [45]. Narratives were thus audio-recorded and transcribed verbatim by interviewers, replacing the identity of the respondents with alphanumeric codes [16].

### 2.3. Data Analysis

As first step, a qualitative analysis was carried out, following the Framework Analysis Technique phases [46]: reading of the transcribed narratives; identification of macro-subcategories/themes; indexing-labelling; construction of thematic chart; and interpretation of contents [47]. Also, a thematic content analysis was carried out [48], starting from the questions included in the interview/topic guide. This was built as preliminary framework, based on theoretical-based categories relevant to the topic to be studied, and taken from the literature and experience of the researchers [16,49]. Statements from the interviews were then broken down within thematic charts (with cases/interviewees in rows and categories in columns), which were provided as Microsoft Excel 2019 (Microsoft Corporation, Washington, USA) sheets [40]. A manual qualitative analysis was carried out without using a dedicated software, as also allowed by literature [50,51], and as deep evaluation process allowing to become familiar with narratives. In this respect, the preliminary conceptual framework was of help, in addition to the cell color-coded process, by means of Excel tools for grouping data based on the color assigned to each category/theme [52]. As a second step, some qualitative dimensions were quantified [53,54] and elaborated using Microsoft Excel 2019 (mainly bivariate analyses). 

For the qualitative analysis, the following main categories/themes were examined: PCA absence and reasons (e.g., high cost, fear, privacy); PCA presence and reasons (e.g., health problems, widowhood); characteristics of PCA presence (e.g., cohabitant/in house, hourly/daily-nightly, recruitment, housing arrangement, employment contract); problems for paying the PCA; communication and satisfaction with PCA. For the description of the sample the following dimensions were also examined, in addition to socio-demographic aspects: four levels of functional limitations, as number of daily living activities/functionalities respondents were not able to perform (mild = no activities “not able”; moderate = one–two; high = three–four; very high = five or more); mobility (in the home, and outside with help); economic situation as monthly income brackets (up to 600-over 2500 EUR). Some absolute values are presented in the Results section without a reference table, as simple frequency of labels in selected answers [16], related to particular details regarding the PCA and when referred by few participants (e.g., why PCA is hired, housing solutions, work contract and cost, financial problems for paying the PCA, communication and satisfaction). 

Moreover, a comparison regarding PCAs vs. DHHs is proposed, by considering some their characteristics (gender, nationality, and main overall tasks, e.g., cleaning the house, washing the laundry, shopping, buying drugs, preparing food, bathing or showering, dressing/undressing, taking medication), and some characteristics of respondents who benefit from such supports (gender, age, level of physical limitations, mobility in the home/outside the home, monthly income in EUR). It has to be clarified that, whereas for PCAs a dedicated section has been provided in the questionnaire/topic guide, for DHHs information has been collected generally as a possible support for performing the several activities of daily living, thus without specific in-depth questions, but only collecting details when spontaneously reported by interviewed older people in this respect. 

Some significant verbatim quotations, from the narratives, were finally included, as statements supporting and integrating the overall analysis [55,56]. Each quotation was labelled with the first three initials and progressive interview number (1–40) of the respective region (LOM = Lombardy; MAR = Marche; CAL = Calabria). A further code, regarding rural/urban site, has not been provided as it could be a potential identifying information of respondents. A few editing of original quotations was provided/necessary, with the aim to facilitate their comprehension, assuring the maintenance of meaning. For this purpose, not relevant omissions were put within round brackets, and some words/terms were added within square brackets, in order to help understanding of quotations’ texts. In the analysis, some territorial differences (regional/urban-rural) are also included, and Lombardy is also identified as North, Marche as Centre, and Calabria as South/Midday. Further details on the methods (setting, sampling, participants, data collection, measures, and data analysis), can be found in a previous publication [16], from which the Section “Materials and Methods” has been partly adapted.

### 2.4. The Analysis Map

Table 1 presents categories, labels and quantitative items analysed in this study. Socio-demographic characteristics of both older respondents and PCAs, and regional and urban/rural dimensions, are not included in this map, as well as some aspects (only qualitative and not quantified) regarding the comparison between PCAs and DHHs. 

## 3. Results

### 3.1. Sample Characteristics

With regard to the whole sample of 120 participants in the main IN-AGE study [16], mainly people aged over 85 (56 units, 47%), women (90 units, 75%), with an elementary education level (55 units, 46%) and widowed (88 units, 73%) were found. Also, a greater mobility outside the home (than only inside), although with help, emerged (72 units, 60%). Concerning physical limitations, respondents reported both mild and very high difficulties in carrying out the various activities of daily living, and the majority (90 units, 75%) referred at least one activity they were unable to perform. A monthly income within 600–EUR 1500 (89 units) was overall referred. 

With regard to the sub-sample of participants having the support from a PCA (*n* = 27), 14 with a cohabiting assistant, and 13 with daily and/or nightly care were found. This distribution confirms the characteristics mentioned above for the whole sample, apart from a worse physical status of older respondents, due to a mobility mainly inside the home, and to a prevalence of very high level of functional limitations. Moreover, PCAs are more present in the South, and with similar values in both urban and rural sites (Table 2).

### 3.2. Why PCA Is Absent

Interviewees without PCA refer various reasons in this respect (Table 3).

Above all, a number of economic reasons emerge (i.e., excessive cost in about half of the cases, especially in rural sites), but also the fact that a PCA is still “not necessary” (41%, especially in urban sites), and the lack of trust for fear of interference and mistreatment (33%, with similar values between sites). Furthermore, although to a lesser extent, the need to preserve one’s freedom/privacy at home also acts as a deterrent to finding the support of a PCA (17% overall, 19% in urban areas). Further reasons point out the lack of space in the house, and the different culture, as possible obstacles. In the regional comparison, the cost is of primary concern more in the South and in the Centre, where also no need and the lack of trust/fear about PCAs are referred with greater evidence. In the North, the lack of space in the house, and the related need to maintain one’s own domestic privacy, are more highlighted.

From narratives details on reasons for excluding PCA as caregiver emerge. 

As for high cost, some respondents generally refer they cannot afford a private personal carer, due to an insufficient income, whereas others more specifically even “do the math in their pockets”, to argue they cannot.

*PCAs want 1400 EUR per month. I do not have such a high pension.*

(CAL_37)

*I have a pension of about 1200 EUR per month.* *If I hire a PCA, then I have no further money for the other necessities.*

(MAR_4)

The statement “still no need” often occurs among respondents who believe they are still in good health, and thus they prefer to be (at least sufficiently) autonomous for as long as possible.

*I do not have the PCA, because I have always managed myself without this support, I would like to continue in this way until the last day of my life.*

(MAR_1)

*With a PCA it would seem to me that I get older. For the moment, even though with a little help, I can go on.*

(MAR_16)

Some interviewees do not trust PCAs, and generally mention that they have heard too many negative rumours about them. Other seniors are more drastic and even report fears of being abused and robbed, as they have sometimes read in the newspapers or heard on the TV. Consequently, it is preferred to remain alone but “not at risk”.

*I am afraid (…), today we read in the newspaper that PCAs even beat the seniors and took their money away.*

(MAR_23)

*PCAs seem all good and helpful, but then they are different. I prefer to be alone. Loneliness is not the best solution, but it avoids further problems.*

(CAL_20)

Lack of trust is sometimes due to a previous negative experience (own or of others) in this respect.

*We hired a woman [PCA] for assisting me when I was sick. At night she did not look at me if I felt bad.*

(CAL_19)

*PCA who supported my mother was a tragedy! She emptied her house!*

(LOM_37)

No strangers in the house, due to privacy issues, is the reason indicated by respondents who want to preserve their freedom. In fact, it seems essential not to give up one’s “single” habits.

*I do not want anyone in my house. I do not accept strangers.*

(CAL_21)

*I prefer to be alone. I have my own television. I hear my music (…). After a while, a stranger annoys me.*

(LOM_20)

The hypothesis in particular of having a cohabiting PCA, creates a lot of perplexity.

*I was thinking to hire a PCA, but I do not want a woman here all day! I need a support just for the night! To have a cohabitant caregiver bothers me.*

(LOM_36)

*No, a steady PCA no (…). I love my freedom.*

(LOM_13)

Also, a too little house, and thus the lack of sufficient space, can affect privacy and consequently discourage hiring a cohabiting PCA.

*I do not know where I could host a private assistant! I should change [apartment].*

(LOM_34)

Three interviewees refer to a different culture/language as a barrier for hiring a PCA.

*No, No (…). If the PCA is foreign no, I do not want her!*

(CAL_35)

*A stranger? In case of need I prefer to move to a care home.*

(MAR_38)

*PCAs only speak their language!*

(CAL_36)

### 3.3. Why PCA Is Present

Only 13 respondents reported a motivation in this regard. In five cases, the need for a PCA becomes greater in the presence of health problems

*I have health problems, in particular incontinence problems, thus I need a PCA.*

(MAR_24)

In two cases it is referred that a PCA is hired to have only a nightly monitoring, due to health problems and also to feel safer.

*I need [PCA] most at night, because I do not feel sure like getting up by myself. I cannot longer be alone at night.*

(CAL_25)

In three cases, a PCA becomes urgent as a result of falls and related fractures of seniors.

*I fell and I broke my ribs, I was hospitalized and then my daughters decided to hire a PCA for supporting me (…). And I am fine with her!*

(LOM_33)

A couple of male respondents reference widowhood, and thus remaining alone is a reason leading to the assistance of a private carer.

*It has been three years since my wife died (…). Since there (…) a PCA was hired.*

(CAL_24)

In one case, a PCA was hired when a daughter could no longer assist the mother because she became sick.

*One year ago, there was my daughter. Then she was sick and could not take care of me anymore. Then a PCA was necessary.*

(CAL_25)

In some cases, current PCAs are “ex-assistants” of spouses, or of deceased relatives (brothers/sisters). Thus, previous positive experiences act as a guarantor for further commitments.

*[PCA] Is a woman who has lived with me for a long time. She assisted my wife.*

(CAL_24)

*This PCA assisted my sister.*

(MAR_34)

### 3.4. PCA Presence and Characteristics

#### 3.4.1. Gender, Age, Nationality of PCA

Among PCAs, 25 women and only two men (in Calabria region, urban sites) were found, and above all foreigners. Over half are indeed from Eastern Europe (e.g., Georgian and Ukraine, mainly in rural sites), and six from extra-Europe (mainly Filipino, only in urban sites). With regard to regions, extra-European PCAs are present almost exclusively in Calabria (especially from the Philippines), whereas Ukrainian assistants are reported almost exclusively in Lombardy, and Georgian only in Calabria. In Marche region, the nationality from Eastern Europe is more varied. It is to highlight that six PCAs are Italian (three in Marche and three in Calabria, four rural and two urban), and due to this circumstance, the term PCA was used in our study, instead of MCW. Moreover, the overall age is between 45 and 65 years, and the youngest are in Calabria (45–60 years) (Table 4).

#### 3.4.2. Cohabitant and Hourly PCA

Among the 27 cases with PCA, both cohabitants/in-house figures (52%, about 70% in rural sites) and hired “by the hour” (48%, 64% in urban sites) emerged. In the second case, it is above all an intensive daytime support of at least 28 h a week (22%, 36% in urban sites), but also only intensive night supervision, or even both possibilities if necessary (Table 5).

Furthermore, in Lombardy only cohabiting PCAs are references, whereas in Calabria and in the Marche the “hourly” mode is more widespread: only daily or only nightly especially in the South, and above all daily/nightly combination, when necessary, in the Centre

From narratives emerged that cohabitant PCA is almost always present. She/he works indeed usually six days out of seven a week.

*She is with me for six days, one day a week she goes home. She supports me day and night.*

(CAL_25)

In three cases, cohabitant PCAs assist even for the whole week, without a day off. These are particular situations where respondents have serious functional limitations, and low family support.

*PCA helps me to take a shower. PCA prepares food for lunch. If I have to buy something I go out with my sister or with the PCA. She is always here with me!*

(CAL_10)

In one case, a cohabitant PCA with no day off during the week can, however, take a full month’s vacation a year to return to her country.

*PCA stays here night and day. Everyday. She has no days off but one month a year she returns to Georgia and then returns here. That is okay for her.*

(CAL_14)

Sometimes seniors suffer when PCAs benefit from their day/hours off/free, and thus they seek the company of a relative or a neighbour/friend.

*She has Thursday afternoon and Sunday afternoon free. However, when she is not here, I am not alone, there is always someone [friend] with me.*

(CAL_23)

*She has a day off. In this case, I am with a niece or a neighbour.*

(CAL_39)

Sometimes, when the cohabiting PCA has her day off, the children provide care for their parents.

*On Sundays [when PCA does not work] my son cooks for me.*

(MAR_20)

From narratives we know that also daily hourly PCA is very present, e.g., every morning, including or excluding Sunday. Overall, this is an intensive day care for at least 28 h a week, but even much more in some cases.

*She comes every day in the morning, except Sunday, from 8.00 h in the morning, to 15.30 h in the afternoon.*

(CAL_24)

*She comes every morning at 8.30 h, and leaves at 12.30 h. On Sundays she also comes to help me in dressing, because I cannot by myself, and if I need something else.*

(CAL_8)

The nightly hourly PCA (three cases in Calabria) “oversees” all nights, except for one case of five nights a week, and usually operates from 20.00–22.00 h in the evening, until 7.00 h in the morning.

*I have a PCA for the night only. I manage by myself during the day. She comes in the evening at 20.00 h, and in the morning at 7.00 h she leaves.*

(CAL_38)

*PCA comes in the evening at 22.00 h, and remain until 7.00 h in the morning, for five days a week. She does not come on Saturday evening and Sunday evening. I gave these rules because it is right for all workers to have a day off.*

(CAL_1)

In one case, a nightly male PCA complements the daily female PCA. A female respondent preferred indeed a male assistant for the night “to feel safer”.

*In addition to my female private carer during the day, I also have a man for the night. The woman goes away in the evening and a man comes, because he gives me more security.*

(CAL_12)

There are also five cases (four in the Marche, one in Calabria) with a combination of daily/nightly assistance, if necessary (the daily PCA remains also at night if needed, and nightly PCA provides also some tasks in the day when required).

*She comes every day, she does everything, two or three hours a day, for what is needed. Then, in the evening she comes to sleep with me in the night, if I need it.*

(MAR_37)

#### 3.4.3. Recruitment of PCA

No respondent reported having searched and found the PCA through advertisements in newspapers, trade unions, or municipal boards. PCA was often recruited through family members, e.g., children, grandchildren, and siblings (44% of cases, mainly in rural areas). Other less frequent recruiting methods (own contact—of respondent, relatives -, friends, “word of mouth”) seem to be more used in urban areas. Furthermore, in all three regions PCA was found above all by the family (children), but also by friends especially in the Marche, and by own contacts mainly in Calabria (Table 6).

From the words of participants, more details emerged. When PCAs are found through direct contact of seniors or family members, they have usually been previous personal assistants of acquaintances, friends and relatives, who guarantee their good care work.

*[Current] PCA assisted the father of an acquaintance of ours. She seemed like a trustworthy, good person to us.*

(MAR_27)

With regard to six Italian PCAs, they are well known in the area, as they have lived there for many years, or are also relatives of the cared for.

*She has lived here for 15 years, with her husband.*

(MAR_37)

*She is a niece of my husband. She lives nearby.*

(CAL_38)

Moreover, “structural” problems, related to the recruitment of PCAs in peripheral/rural contexts, are reported, since these are zones difficult to reach without a car.

*This place [Lombard rural municipality] is isolated, and PCA are often without cars. It is difficult to find a personal assistant who can move independently.*

(LOM_27)

#### 3.4.4. Housing Arrangements

No respondent reported having adapted/modified home environment, in order to accommodate cohabiting or nightly PCA (22 case on the whole). In 20 cases, there is indeed a dedicated room available for this assistant (sometimes also with personal bathroom and TV). Usually, the room for the PCA is the previous bedroom of children or other relatives who are no longer living with the cared for.

*PCA has her own room, which was my daughter’s bedroom.*

(CAL_1)

*PCA sleeps in my mother-in-law’s former bedroom, and has a separate bathroom.*

(LOM_33)

Only in two cases the PCA sleeps in the senior’s room (Marche and Calabria), and in one of these, even in the same bed (Calabria), since the cared for thinks that sleeping together makes her feel safer.

*We sleep in the same bed, PCA has not a separate room. I gave her a piece of furniture in the bedroom for her clothes and so on. She is very protective and takes care of me at night. During the night she checks if I am adequately covered with the blanket.*

(CAL_23)

#### 3.4.5. Work Contract and Cost

Regarding the type of employment and related cost, few seniors reported precise information. Overall, when referred, regular employment contracts in 11 cases emerged (especially in the North and urban sites), and irregular employment/verbal agreements in 10 (especially in rural sites in Calabria).

*PCA has a regular contract, I pay her every month, including legal INPS [National Institute for Social Security] contributions.*

(CAL_10)

*She has been here with me for 7–8 months and has no contract. She works in “black” [verbal agreement]. PCAs have no documents.*

(CAL_40)

The monthly cost ranges between EUR 300 and 1150, in addition to room and board in case of cohabitant PCA. It should be noted that the overall average range is EUR 700–1000 per month, and 700 regards also some cases of non-cohabiting but intensive daily PCAs, whereas EUR 300–400 per month represents the wage of PCAs who are illegally hired with informal verbal agreement, and usually requested only for the night or minor/not hard daily tasks, also due to financial difficulties of older people (e.g., five cases in Calabria, four urban, and one rural).

*We have a verbal agreement, and PCA receives 300 EUR per month. I take 550 EUR for my pension. I cannot give her more than this.*

(CAL_8)

*I have a PCA only for the night. Every month, when I take my pension, I give her 300 EUR.*

(CAL_38)

### 3.5. Financial Difficulties

Financial difficulties in paying the PCA indirectly emerged when it was asked to report possible overall problems for current expenses in the last year (for clothes, utilities, specialist visits, drugs, diagnostic tests, maintenance and house repairs, and PCA). Only 13 respondents with PCA mentioned economic issues in this regard. Of these, in seven situations, the salary of the seniors was considered sufficient.

*I can pay for her with my resources, attendance allowance and work pension.*

(MAR_20)

*No, no, I have no difficulties. I thank God, I do not make sacrifices to pay a PCA.*

(CAL_39)

However, in one case there is the financial support of a child, when needed.

*When I need, my son helps me, he pays something too [for the PCA].*

(MAR_27)

Conversely, in six situations problems for paying the current PCA were reported, in addition to some sacrifices in order to have such a support.

*Well, the pension is that! I give up some things [to pay the PCA], for example to buy a dress. I wear what I have, I cannot buy much. I only buy the essential and the necessary.*

(CAL_8)

### 3.6. Communication and Satisfaction

Communication problems with current foreign PCAs (or MCWs), seem to emerge only in five cases (Lombardy and Calabria), especially due to the imperfect knowledge of the Italian language, even though these are initial situation improved over time.

*I have communication problems with the PCA, but we try to understand each other. She does not speak Italian very well.*

(CAL_39)

*At first, it was a bit more difficult. Now she speaks better but not yet well. She does not use articles and prepositions, but I try to understand her.*

(LOM_3)

The satisfaction with the assistance of a PCA is essentially almost total (25 out of 27). Interviewees reported they feel comfortable with PCA, and stated that this support as indispensable, a positive experience to be recommended.

*She is very good, a positive experience which I would also recommend to others.*

(MAR_24)

*She is my help! She is good, knows how to cook, she is a clean person!*

(LOM_40)

*She is like a mother for me. I am fine with her (…). It is like having a relative.*

(CAL_14)

### 3.7. PCA vs. DHH 

By comparing overall some results regarding PCAs (23% of cases, 27/120), and DHHs (37%, 44/120), some differences, in relation to some characteristics of the interviewees, emerge (albeit within the limits of less information collected for the latter, as mentioned in the Methods) (Table 7).

There is in fact a generalized prevalence of female seniors cared for (this concerns the entire IN-AGE sample), but especially if DHH is present (82%) compared with PCA (67%). Furthermore, a worse context emerges for older people with PCA, who denote greater frailty and from different points of view: higher age, prevalence of serious physical limitations, and mobility, especially only at home. However, there is an overall, slightly better, income situation when a PCA is hired, although there is an overall concentration of older participants in the group having EUR 600–1500 per month.

Despite these differences, from interviews (and when details were referred for DHHs), some contact points between DHHs and PCAs can be identified in the gender, almost totally female (only two male PCAs and one male DHH in Calabria), and in the nationality, mostly foreign (21 cases among PCAs, and 11 among DHHs). With regard to PCAs, respondents state as follows:

*He is Filipino, and the lady is Romanian.*

(CAL_12)

*PCA is stranger, is Filipino.*

(CAL_1)

*She is from Romania.*

(CAL_25)

*PCAs are all Georgian and Ukrainian.*

(CAL_40)

Similarly, with regard to DHHs, respondents state as follows:

*She is from the East. She is a wonderful person.*

(MAR_4)

*For housekeeping and cleaning I have a woman, she is a Filipino.*

(CAL_13)

In the testimonies collected, other details highlighted further similarities between the two figures. The tasks performed by the PCAs are cleaning the house, washing the laundry, shopping, preparing food, bathing or showering, help in taking medication. These personal assistants provide “everything” is needed,

*She prepares food, cleans the house, washes the laundry, gives me the medicines.*

(CAL_14)

*She does everything. He cooks because I cannot stand for very long time to cook. She does the laundry, provides cleaning and shopping, she takes care of the management of the home. She helps me with the bath and dresses me. She also helps me in taking drugs.*

(CAL_25)

*She accompanies me if I need to go out, cooks for me, cleans all, keeps me company, runs external commissions, many things!*

(MAR_39)

Concerning in particular nightly PCAs, their main tasks are to supervise/monitor and make company to older people when they sleep, and to intervene especially in case of illness.

*PCA comes at night, I keep her for company, in order to not be alone. She helps me in case I need to call a medical doctor, if I am sick.*

(CAL_38)

Interviewees also confirm the same tasks for DHHs, who usually mainly deal with house cleaning, but (similar to PCAs) they often also help with shopping, cooking meals and various other commitments, e.g., withdrawal of prescriptions from the General Practitioner (GP), and purchase of drugs. With regard to shopping, the support of DHH is important, especially to bring home what has been purchased, which is crucial when older persons live in a building with stairs and without a lift.

*I cannot go shopping, because even a kilo is heavy for me. I cannot take it anymore. For this I have a DHH.*

(MAR_13)

DHHs also sometimes help with bathing and dressing, although they are not referred to as hourly PCAs.

*The girl who cleans my house, also gives me a shower, because I cannot do it alone anymore.*

(MAR_22)

*She [DHH] is so good. I really like her. She helps me a lot to take a bath, to keep me in order.*

(MAR_5)

*I have a person who comes in the morning to clean the house, fix my bed, do the essentials for me. This lady helps me also with the bath.*

(CAL_35)

Seniors can rely on DHHs even if they do not feel well.

*I take a bath with DHH, and when I feel bad, I call her. She has been helping me for housekeeping for many years.*

(MAR_32)

However, a lower hourly commitment for a DHH, than PCA, is detected. Home helpers never work at least 28 h per week (as already highlighted for PCAs).

*I have a woman who comes once a week. She works three hours a week. She is a Filipino.*

(CAL_13)

*I have a Muslim girl. She comes once or twice a week, to do cleaning and more.*

(LOM_12)

Sometimes respondents do not even feel the need for a PCA because DHH meets all needs; thus, the help is more than enough.

*I do not think about her [PCA] right now. Now I have a DHH. She is good. If I ever want a PCA, practically, I already have her!*

(MAR_22)

In some circumstances, a DHH may even become PCA within the same family, especially if an older relative becomes ill, and needs more hours of assistance.

*This lady [current PCA] did heavy cleaning for me and my wife. When my wife got sick in 2013, the lady intensified her support, and became her PCA, and then mine.*

(CAL_24)

Conversely, in other situations, a PCA may become DHH in the same family, e.g., in case she was caring for the wife of a senior who becomes widower, but needs less assistance than his spouse.

*I have a DHH who was the PCA of my wife. She now continues to come for me, for cleaning the house, ironing my pants, shirts and so on.*

(CAL_9)

## 4. Discussion

### 4.1. PCA Absence and Presence 

The aim of the study was to explore role and characteristics of PCAs of frail older people with functional limitations, ageing alone in place in Italy. In this respect, this country represents a family-based model of LTC, since family caregivers usually support their older frail relatives at home [57]. However, following the decreasing availability of family care arrangements, PCAs represent a bridge to ageing in place and reproduce familism [58]. The Italian elder care model has thus moved from being “familism by default” to “implicit-supported familism” [59] (p. 174), and the “woman-in-the family” has been often complemented by the “migrant-in-the-family” [60] (p. 65). 

However, as emerged from our results, PCA cannot be always present, due to various reasons, such as lack of need and the feeling of being autonomous, fear of mistreatment, need for freedom and privacy, but mainly due to economic reasons of the cared for and respective families, since the related cost is not affordable for all. Previous studies underline socio-economic inequalities in accessing the private care market by older people, and particularly the cost represents a financial barrier for many [57,61]. According to DOMINA [34], when seniors are dependent due to functional limitations, the need for assistance increases, and consequently the economic cost of private care. In this case, very few older people are able to afford the assistance provided by a PCA, relying only on their pension (about 8% of retirees, and only 4% if a trained worker is required). Regarding the fear of abuse, the previous literature showed older people reporting being mistreated by privately hired PCAs (mainly physical and psychological abuse) [62]. In such contexts, the housing of the older adults living alone could really become a dangerous place of loneliness and neglect [63]. The cost represents a barrier, especially in rural sites and in the South and Centre of Italy, which are effectively areas less wealthy than urban sites and the North [64], respectively; the fear for being abused by a PCA mainly in the South and in the Centre emerged, probably due also to a greater trust on the family care in these regions [16].

Our findings further highlighted that when the PCA is conversely hired, this depends above all on health problems of the cared for, their falls and consequent fractures, in case of widowhood and/or when the older person does not want to stay alone at night, thus requiring monitoring. Indeed, some authors define the functional status of the person in need of care, and the level of disability, as predictors/enabling factors for employing a PCA in Italy, in addition to available income and receiving ca are allowance, e.g., IA [57]. Moreover, ISTAT data [65] indicate that the share of older people who have the support of a PCA, increases from 6% to 28% when severe functional limitations intervene. It should be emphasized that, in our study, a previous positive or negative experience with PCA regarding friends or relatives, seems sometimes crucial and can influence the willingness/choice to hire or not a personal assistant. 

In a regional comparison, more PCAs in the South were detected, and this finding is also confirmed by the literature [18], which highlights how public home services are more widespread in Northern regions, and less in the South of Italy, and this imbalance is partly “balanced” by the use of PCAs, with 7% of older people living alone supported by PCAs in the North, 9% in the Centre, and 10% in the South. Moreover, the same source reports that the presence of PCAs who assist on a daily basis is slightly higher in the South (6%) and in the Centre (7.5%), than in the North (5%). Thus, where the need for care is greater and the presence of public services is scarce, the recourse to the private care market increases: a situation that puts in evidence a territorial inequality in the approach and capacity to satisfy/meet the needs of frail seniors living alone.

### 4.2. When PCA Is Present 

In our study, PCAs are almost all women, except for two men in Calabria region, mainly from Eastern Europe, and aged 45–65 years. Other authors [57,66] confirm the abovementioned general characteristics. In particular, DOMINA [34] highlights the presence of male PCAs as a minority component growing in recent years. According to this source, men represent on average 11.3% of total regular domestic workers, with a greater incidence among DHHs (14.5%) than PCAs (7.8%). In particular, in our study all and half of total are from Eastern Europe, in the Lombardy and Marche regions, whereas the extra-European component is almost present exclusively in Calabria. This is probably depending (at least partly) on the greater geographical proximity of the Central-Northern regions to Eastern Europe, that is the area of origin of the vast majority of PCAs [34]. Moreover, in our findings the presence of six Italian PCAs is concentrated in Marche and Calabria regions, and this is probably linked to the overall economic and labour market difficulties and lack of work opportunities, especially in the South of Italy, as circumstance leading to seek job opportunities in the sector of caring and domestic work [34].

Our study also found both cohabitant/living-in and hourly PCAs, in a quite similar proportion. Moreover, if the cohabitant PCA is almost “always” present, i.e., living with the cared for and working usually six days out of seven a week, nevertheless the hourly PCA is really “very” present, especially with an intensive day care for at least 28 h of work per week, but also much more when needed. Also, the need to have a substitution, when cohabitant PCA has a day off, and cases of intensive assistance for seven days a week, emerged. DOMINA [34] puts in evidence data partially different for Italy, with a minority of living-in care workers (34%), and a greater 66% hired on hourly basis, with cohabitation requiring a greater number of working hours, on average 38 h, against 20 for non-cohabitants. Other authors indicate that families hiring PCAs expect them to care around the clock, i.e., 24 h each day [67], whereas they officially (by regular employment contract) should work up to 54 h per week and up to 10 h per day [61,68]. Our findings further stressed the existence of combination of daily/nightly assistance, and the importance in particular of a supervision during the sleep. According to Martinelli and colleagues [69], the presence of a PCA, especially at night, is indeed an element of strong reassurance, also regarding the fear of intrusions and thefts. It is necessary to highlight that, in the interviews, “in house” modality prevails in rural sites and in Lombardy, whereas the “daily” and “nightly” ones are slightly more common in Calabria and in the Marche, mainly in urban sites. In this respect, De Rossi [70] indicates that, in rural sites, the consequences of migration to urban areas of younger relatives/children seems to impact on the greater necessity of cohabitant PCAs. Moreover, especially in the South, even though the need of support is greater because respondents are older and frailer, a PCA on hourly basis must be “enough”, due to a less financial availability. ISTAT [71] underlines that in the South of Italy, where social pensions (provided to those who are in poor economic conditions, aged at least 67 years) are more common than work pensions, 24% of retirees belong indeed to the poorest quintile of income, and only 16% are placed in the richest one. Conversely, in the North-Centre these values are reversed and about 16% and 23%. It should be considered that, on the whole, cohabitation is a common housing solution for migrant PCAs when just arrived in Italy, whereas larger available support networks have permitted, in more recent years, a higher housing autonomy [72], especially when hired on hourly basis.

When referred by our respondents, regular working contracts in 11 cases emerged and irregular/verbal agreements for undeclared work in 10. According to DOMINA [31,34], in 2017 60% of irregular figures in Italy was estimated, and over 50% in 2020. Other sources [19,61] estimated only 40% of foreign PCAs regularly hired in 2020. Italian families have become “domestic employers”, but often they have not a complete/precise knowledge of legal norms occurring in the private care market, and when also low economic resource need to be considered, they rely on irregular/undeclared PCAs to reduce costs, exposing their loved ones to possible inadequate assistants. Thus, due to lacking public welfare and LCT provisions (especially in-kind services), and under a “spending review” regime, the Italian State has favoured a “family welfare”, mainly providing direct monetary transfers and delegating the management and responsibility of care to families [34]. Further authors stress this issue, by stating that in Italy the overall presence of PCAs is allowed by the existence of a considerable unregulated labour market [58], that in turn is supported by cash for care allowances (e.g., IA), with families acting as employers and hiring such care workers often outside the formal economy. In Italy, PCAs, especially when are migrant workers with precarious working conditions, face indeed several inequities in accessing to labour protection [73]. Since there are strict immigration laws in this country, and a regular employment contract is requested to obtain a residence permit as well as to access to welfare services, various ad hoc regularizations processes of undeclared PCAs with a migration background have been provided [58,74], mainly as “ex post facto approval system” of migrant policy [59] (p. 171), with the latest occurring in the period June–August 2020, and recording about 122,000 applications [75]. 

Our study also pointed out the existence of a link between irregular working condition and low salaries in some cases. The monthly cost ranges EUR 300–1150, and PCA receives EUR 300–400 per month when hired illegally/with verbal agreement. However, a low salary occurred also when PCA is requested only for the night or for minor daytime tasks (only a few hours a day for house management and care of the senior). In the latter case, the low remuneration is essentially proportionate to the type of function performed, and therefore does not seem to configure a situation of “exploitation”. As stressed also by previous authors [57], in many countries, not only in Italy, PCAs have often unregulated jobs (as parallel markets of private care) and are usually low-paid, with unmonitored care quality. DOMINA [34] reports that regular annual costs for a PCA vary from 2000 EUR, for five hours a week, to almost 15,000 for a 54-h care with cohabitation. According to further sources [61,68], PCAs living-in with formal contracts should be officially paid about EUR 1000–1200 per month, following their skills and tasks. In some cases, however, low wages of PCAs allowed relatively low-cost private care services, as substitute or complement of public ones [58]. Especially in rural sites in Calabria, no regular contract was referred by respondents. Moreover, in five cases in this region (four urban and one rural) the PCAs receive EUR 300–400 per month, when there is only a verbal agreement, or the support is requested only for the night/few daily hours. This finding once more confirms the fewer economic possibilities to hire a PCA in these areas, but despite this, the recourse to this personal assistant seems sometimes necessary, at least for a reduced period of time and without a legal employment, since this permits lower wages. It is also worthy to mention that in our study the cost represents an overall major issue, a central node, both for those who cannot afford PCAs, but also for those who already have such a support but sometimes report economic problems for payment of a private care. In six situations respondents report indeed serious difficulties for paying the current PCA, with consequent waivers. Thus, in Italy, on the one side a widespread availability of “economic benefits” favors the use of the private care assistance market, but on the other side these financial allowances do not cover the whole costs of PCAs [67]. 

Furthermore, the PCAs in our findings were never recruited by the interviewees through advertisements, trade unions, or municipal registers, but rather through family members, especially children. This seems to indicate that currently, seniors and families, probably do not trust on the usefulness of such non-familiar channels, but rather they rely and trust on the family cooperation in this respect. It should be highlighted that often, citizens do not know the existence in particular of municipal registers, to consult in order to find a qualified PCA, and also such registers are not still provided in every city. Some sources [76] indicated that few private agencies were provided in Italy, in order to put in contact families and PCAs, usually acting for a fee, thus families rarely accepted such a solution, for instance in absence of other recruitments channels. On the whole, a wider question of lacking information on recruiting/accessing available support services emerges, especially when this is online and implying the use of internet, due to scarce digital skills of cared for and relatives, often both older [77]. Also, our results indicate that the PCA was often found through family members, especially in rural and North-Central areas. The use of information from own acquaintances especially in Calabria emerged. In this respect, Martinelli and colleagues [69] put in evidence greater ties in the South of Italy, generally existing also with regard to neighbours and friends. Moreover, recruiting a PCA can be sometimes particularly difficult in rural/peripheral areas, since these are complicated to reach if a car, or someone who can act as a “driver”, are not available.

Our results also indicate that no changes in the home were provided, in order to accommodate the live-in or night PCA. Therefore, in the decision to resort to the help of the “personal assistant”, the possibility of having sufficient space and of not having to adapt/modify the structure of the environment, seems to impact positively. The problem of having scarce space in the house emerged in fact as reason leading to the exclusion of hiring a PCA, in particular through cohabitation. Some literature underlines housing as a fundamental short-term and long-term question for migrants [78], who try to construct their domestic spaces, and to feel “at home” in receiving societies [79]. 

Our interviewees further pointed out some considerations on the relationship cared for-PCA, as for communication and satisfaction issues. Overall, seniors are practically all satisfied with this support, despite a few cases with communication problems, due to an imperfect knowledge of the Italian language, although these were initial situations improved over time, and partly solved. Literature underlines empathy and understanding as crucial aspects for care work [80]. Language and communication questions, cultural differences, in addition to possible discriminatory attitude of some older people, may indeed represent barriers for employing PCAs [81]. In particular ethnicity of PCAs sometimes impacts willingness to hire them for caring older relatives, who in some cases accept help only from certain nationalities. Thus, the relationship between PCA and older people cared for is extremely important for making this care worker being part of the family [67], and recruitment and skill requirements of PCAs are aspects requiring particular attention [81].

### 4.3. PCA vs. DHH

From our findings some differences and similarities, between PCAs and DHHs, also emerged, which overall denote an increasingly “blurred” border in this respect, and to some extent redesign a more updated profile of these workers. Both are private family assistants, frequently employed by the families of older people, especially when living alone. PCAs (cohabiting with the assisted person or hourly hired) are usually dedicated to personal care, possibly also in combination with domestic tasks. DHHs are mainly engaged in housekeeping and other household tasks (e.g., washing, shopping, preparing meals). Apart from these definitional and other differences (e.g., the presence of the PCA seems to exist especially when the interviewees are older, with a lower level of autonomy, and with a higher income), both are mainly foreign women who sometimes provide very similar functions (home and personal care), although a different hourly commitment emerges, with at least 28 h a week (even at night if necessary) for the hourly PCA, and about 3–6 h a week (1–2 days a week) for the DHH. Also, authors from other countries [82] highlight that, in recent years, the boundary between domestic and care work is overall less clear compared with the past. The boundary between these two types of private family assistants is so blurred in our study that often DHHs become PCAs and vice versa, within the same family or in different families. However, more domestic workers than personal assistants emerged (44 cases vs. 27), probably due to the lower cost of the former, related to a lower number of hours worked per week, thus implying a less-demanding presence in the home, and consequent greater freedom and privacy protection for the seniors. According to DOMINA [34], in 2019 in Italy, regular PCAs were 48% and regular DHHs 52%. This source states that the average cost of a PCA is not always sustainable for a pensioner, if compared with a DHH hired on hourly basis, even though the situation improves when IA is available, since it adds over EUR 6000 to the annual income. However, data regarding the last 10 years, indicate that DHHs (not only for older people) have progressively decreased (−32% since 2012) and PCAs have increased (+11% since 2012). This opposite trend is probably due to a greater need of care for older people (and to a decrease in the support provided by family members), whereas the economic crisis has generally generated a renunciation to domestic workers by many families, especially when seniors are not present. Therefore, if the PCAs/DHHs ratio was 1:2 10 years ago, currently it is near to 1:1. 

### 4.4. Limitations and Validity of the Study

The study has some limitations to be considered. The definition of frailty is based only on people aged 65 and over, living alone without cohabiting family and presenting functional limitations, thus needing support, as supported by some literature (e.g., [1,10]). A cognitive assessment of participants was based only on the information provided by the recruitment channels, in addition to those from respective relatives. The overall low number of PCAs included in the study may be connected also to the exclusion of older people with serious physical and cognitive limitations, who are more often supported by these care workers. In turn, the greater number of PCAs sampled in Calabria, than in Lombardy and Marche regions, seems related also to a greater number of frail seniors aged 85 and over mapped in the former. Moreover, broad income classes were used, since interviewees in few cases referred a monthly punctual income. Finally, percentages values in tables are to be interpreted with caution, since related absolute values are sometimes very low. 

Despite these limitations, in our study the validity in particular of the qualitative analysis was assured, as based on the following criteria: credibility, transferability, dependability and confirmability [83]. The credibility is satisfied by the following aspects: use of a topic guide in great part based on questionnaires applied in previous studies on frail older people [41]; peer de-briefing sessions among expert researchers (e.g., to define protocol, and topic guide); and seminars with several stakeholders in order to discuss first findings. The analytic (not statistical) transferability of qualitative analysis [84] is supported by a deep literature review [85], and analysis of studies on the topic [18], which were used to build the starting framework [17]. The dependability and confirmability of results, as duration of the results by means of replicable Methods, were achieved through a detailed study protocol (approved by a Bioethics Committee), with several indications regarding data collection and analysis [85], as the use of the cell color-coded process. For more details on limitations regarding the IN-AGE study, and validity of the overall qualitative data analysis, additional information can be found in a previous publication [16], from which these aspects are partially taken.

## 5. Implications

PCAs seem indispensable to the Italian care systems, with direct employment by families [59,86] as care solution at home better than moving to a nursing home [67]. Also, the Italian National Recovery and Resilience Plan (NRRP) [87] highlights the importance of proximity care networks, especially for home care [22]. Moreover, in Italy an overall integration model of “tolerance,” seems to exist, where PCAs are socially accepted nevertheless their frequent illegal status [58].

Starting from these considerations, it seems necessary to integrate PCAs in the Italian LTC system, also impacting its sustainability [73,88], within the network of local services, and to provide incentive systems, as follows: fiscal benefits for related expenses [89]; means-based assessment of the IA to identify people most in need of financial assistance [57,61]; and care allowances for regular hiring of PCAs [90]. Irregular employment impacts indeed their working conditions [81], which in turn impact quality standards of care services for older people [58]. It seems also crucial the ongoing education and training of PCAs, including language provision [81], especially with reference to particular nursing and personal care tasks, in order to improve the wellbeing of seniors cared for [30,57]. A first step in this direction has been moved in Italy, since the new National Collective Labour Agreement introduces the quality certification of DHHs and PCAs. The contract provides indeed the possibility to obtain a “quality seal”, by training and accreditation of compliance with the Rule Uni 11766: 2019. This rule has indicated the requirements of competence, ability and knowledge defining a domestic-care worker certified as “quality professional” [91]. However, even though some regions provide such training course [92], in particular with programs until 2027 [22], a national-level system in this respect is still lacking [61]. Moreover, to integrate migrant and elder care policies [81], and to integrate PCAs socially and culturally into the hosting country [58], seem winning strategies.

It is finally to highlight that the phenomenon of PCAs in Italy, especially when they are MCWs, has been greatly affected by the COVID-19 pandemic, thus bringing out their precariousness. Some authors (e.g., [88]) report how some PCAs continued to work in dangerous circumstances, whereas others, especially when informally employed and irregular, lost their job, due to the need for precautionary social isolation to avoid contagion. Older people faced crucial situations, with fewer hours of assistance, but also completely without this support, and sometimes in complete loneliness and isolation, especially those living alone at home. On the whole, during the pandemic, both residential and home care, including PCAs/MCWs, showed crucial limits with meeting LTC needs of frail older people ageing in place [93], and the related urgent necessity to provide overall efficient territorial and home services. In order to reach this goal, it seems important to carry out future research focused on PCAs, integrating experiences of frail older people needing support, their family, and especially migrant carers, as a “triage” to be adequately managed. This in order to explore also developments and evolutions of the PCAs phenomenon related to the effects of the pandemic, in addition to analyses comparing other Italian regions and international contexts.

## 6. Conclusions

Our findings highlight that PCAs in Italy are mainly foreign (MCWs) women from Eastern Europe, often hired with verbal agreements, which cost represent a crucial issue. Despite this, their involvement in caring for frail older people, especially with health problems, victims of falls and fractures, represents a “bridge” buffering the decreasing provision of informal family care and the low/inadequate provision of public home services. Similarities between PCAs and DHHs also generally emerged, with quite same functions but different hourly commitments. Moreover, regional differences put in evidence a higher support of these personal private assistants in the South, where also more irregular work contracts and financial difficulties, in this respect, are reported. It seems thus overall necessary to integrate PCAs in the Italian LTC system, by means of incentive systems for their hiring, and more opportunities of education and training on caregiving of older people.

## Figures and Tables

**Table 1:** Categories, labels, and quantitative items.

Macro-Categories	Sub-Categories	Labels for the Analysis	Quantitative Items (N = Number)
Personal care assistant (PCA) presence	Reasons for hiring	Health problems, falls, widowhood, nightly monitoring	N. respondents reporting each reason
	Characteristics of PCA	Cohabitant/hourly (daily-nightly), recruitment, housing arrangement, work contract, cost	N. cohabitants/hourly N. recruited by relatives (e.g., children), own contact (of respondent, relatives), friends, “word of mouth” N. with/without own room N. regular/irregular contracts Monthly cost in EUR
	Financial difficulties of respondents	Problems for paying the PCA	N. respondents with problems N. respondents without problems
	Relastionship	Communication Satisfaction	N. respondents reporting communication difficulties N. respondents reporting satisfaction
Personal care assistant (PCA) absence	Reasons for not hiring	High cost, still no need, lack of trust/fear, no strangers in the house/privacy, lack of space in the house, different culture/language	N. respondents reporting each reason

**Table 2:** Sample characteristics (absolute values/n).

Characteristics	Regions and sites						Total
	Lombardy		Marche		Calabria		
	Urban	Rural	Urban	Rural	Urban	Rural	
**Living situation (*n* = 120)**							
Alone	23	13	21	11	14	11	93
Cohabitant PCA	1	3	1	2	3	4	14
Not cohabitant/hourly PCA ^1^	-	-	2	3	7	1	13
**Total cases/respondents**	24	16	24	16	24	16	120
**Participants with PCA (*n* = 27)**							
Age Groups (years)							
67–74	-	-	-	-	2	-	2
75–79	-	1	-	-	1	-	2
80–84	-	-	-	1	-	1	2
85 and over	1	2	3	4	7	4	21
Gender							
Female	1	1	1	5	7	3	18
Male	-	2	2	-	3	2	9
Education							
No title	-	-	-	2	-	3	5
Primary school (5 years)	-	2	2	1	4	2	11
Middle school (3 years)	-	1	1	1	-	-	3
High school (3–5 years)	1	-	-	1	5	-	7
University/similar (3–5 years)	-	-	-	-	1	-	1
Marital status							
Single	-	1	-	-	2	-	3
Married but not cohabiting	-	1	1	-	-	-	2
Divorced/separated	-	-	-	-	1	-	1
Widowed	1	1	2	5	7	5	21
Level of physical limitations ^2^							
Mild	-	-	-	1	-	-	1
Moderate	-	-	-	2	1	1	4
High	-	-	1	1	4	1	7
Very high	1	3	2	1	5	3	15
Mobility							
Only in the home	1	1	1	2	6	4	15
Also outside the home with help ^3^	-	2	2	3	4	1	12
Monthly Income brackets (EUR)							
Up to 600	-	-	-	-	1	-	1
601–1500	-	3	2	5	4	3	17
1501–2500	1	-	1	-	3	2	7
Over 2500	-	-	-	-	2	-	2
Missing	-	-	-	-	-	-	-
**Total respondents with PCA**	1	3	3	5	10	5	27

^1^ Daily/nightly regular attendance for at least 28 h a week; ^2^ The level of physical/functional limitations is based on 12 Basic and Instrumental Activities of Daily Living (ADLs-IADLs), two mobility limitations (going up/down the stairs and bending to pick up an object), plus sensory limitations in hearing and seeing. Mild = no activities “not able”, Moderate = one–two, High = three–four, Very high = five or more; ^3^ Respondent is able to leave the house, at least two times a week, only if accompanied or with aids (cane, walker); ADLs: getting into/out of bed, sitting/rising from a chair, dressing/undressing, washing hands and face, bathing or showering, and eating/cutting food; IADLs: preparing food, shopping, cleaning the house, washing the laundry, taking medication in the right doses and at the right times, managing finances.

**Table 3:** PCA absence and reasons, by sites and regions.

Reasons ^1^	Urban		Rural		Lombardy		Marche		Calabria		Total	
	*n*	%	*n*	%	*n*	%	*n*	%	*n*	%	*n*	%
High cost	24	41	20	57	15	42	16	50	13	52	44	47
Still no need	28	48	10	29	12	33	15	47	11	44	38	41
Lack of trust/fear	20	34	11	31	7	19	13	41	11	44	31	33
No strangers in the house/privacy	11	19	5	14	11	31	2	6	3	12	16	17
Lack of space in the house	4	7	3	9	4	11	2	6	1	4	7	8
Different culture/language	-	-	3	9	-		1	3	2	8	3	3
Total respondents without PCA	58	100	35	100	36	100	32	100	25	100	93	100

^1^ More reasons are possible.

**Table 4:** PCA presence: age, gender, and nationality, by sites and regions.

PCA: Gender, Age, Country	Urban		Rural		Lombardy		Marche		Calabria		Total	
	*n*	%	*n*	%	*n*	%	*n*	%	*n*	%	*n*	%
Age range ^1^	45–60 ^2^	--	45–65	--	48–61	--	50–65 ^2^	--	45–60 ^2^	--	45–65 ^2^	--
Gender												
Male	2	14	-	-	-	-	-	-	2	13	2	7
Female	12	86	13	100	4	100	8	100	13	87	25	93
Nationality												
Italian	2	14	4	31	-	-	3	37	3	20	6	22
Foreign ^3^	12 ^3^	86	9	69	4	100	5	63	12^3^	80	21 ^3^	78
Europe	6	43	9	69	4	100	4	50	7	47	15	56
*Georgian*	*2*	*14*	*3*	*23*	-	-	-	-	*5*	*33*	*5*	*19*
*Ukraine*	*1*	*7*	*4*	*31*	*4*	*100*	*1*	*13*	-	-	*5*	*19*
*Romanian*	*2*	*14*	*1*	*8*	-	-	*1*	*13*	*2*	*13*	*3*	*11*
*Albanian*	*1*	*7*	-	-	-	-	*1*	*13*	-	-	*1*	*4*
*Moldovan*	-	-	*1*	*8*	-	-	*1*	*13*	-	-	*1*	*4*
Extra-Europe	6	43	.	-	-	-	1	13	5	33	6	22
*Filipino*	*4*	*29*	-	-	-	-	-	-	*4*	*27*	*4*	*15*
*Sinhalese*	*1*	*7*	-	-	-	-	-	-	*1*	*7*	*1*	*4*
*South American*	*1*	*7*	-	-	-	-	*1*	*13*	-	-	*1*	*4*
Total respondents with PCA	14	100	13	100	4	100	8	100	15	100	27	100

^1^ Some respondents did not report the age of the PCAs (11 cases); ^2^ Only two cases of 30 and 35 years (not included in the range); ^3^ Foreign PCAs are reported in 21 cases (12 cases in urban sites), but they are in total 22 (13 in urban sites) due to the presence of two PCAs for a single case in Reggio Calabria. However, the sum of the European and extra-European PCAs is 21 and not 22; since in an urban case (Reggio Calabria), the nationality was not indicated.

**Table 5:** PCA presence: cohabitant-in house/hourly, by sites and regions.

PCA Cohabitant/in House, Hourly	Urban		Rural		Lombardy		Marche		Calabria		Total	
	*n*	%	*n*	%	*n*	%	*n*	%	*n*	%	*n*	%
Cohabitant/in house	5	36	9	69	4	100	3	38	7	47	14	52
Hourly ^1^	9 ^1^	64	4	31	-	-	5	63	8 ^1^	53	13 ^1^	48
*Daily (at least 28 h per week) ^2^*	*5*	*36*	*1*	*8*	-	-	*1*	*13*	*5*	*33*	*6*	*22*
*Nightly (at least 28 h per week) ^3^*	*2*	*14*	*1*	*8*	-	-	-	-	*3*	*20*	*3*	*11*
*Daily/Nightly (depending)*	*3*	*21*	*2*	*15*	-	-	*4*	*50*	*1*	*7*	*5*	*19*
Total respondents with PCA	14	100	13	100	4	100	8	100	15	100	27	100

^1^ Hourly PCAs are reported in 13 cases (nine cases in urban sites, eight cases in Calabria region), but they are in total 14 (10 in urban sites, nine in Calabria)—*sum of the values in Italic*—due to the presence of two figures for a single case in Reggio Calabria (one for the day and one for the night); ^2^ Daily = even if present 6 days a week; ^3^ Nightly = even if present 5 nights a week.

**Table 6:** PCA presence: recruitment by sites and regions.

PCA: Recruitment Channel ^1^	Urban		Rural		Lombardy		Marche		Calabria		Total	
	*n*	%	*n*	%	*n*	%	*n*	%	*n*	%	*n*	%
Relatives (e.g., children)	4	29	8	62	2	50	4	50	6	40	12	44
Own contact (of respondent, relatives)	4	29	2	15	-	-	1	13	5	33	6	22
Friends	3	21	2	15	1	25	3	38	1	7	5	19
“Word of mouth”	2	14	1	8	1	25	-	-	2	13	3	11
Total respondents with PCA	14 ^1^	100	13	100	4	100	8	100	15 ^1^	100	27 ^1^	100

^1^ In one case no answer was provided (related sums are 13 and 26, respectively).

**Table 7:** Personal Care Assistant (PCA), Domestic Home Help (DHH), and older people.

Some Characteristics of Respondents ^1^	DHH		PCA	
	*n*	%	*n*	%
**Gender**				
Female	36	82	18	67
Male	8	18	9	33
**Age Groups (years)**				
67–74	9	20	2	7
75–79	8	18	2	7
80–84	12	27	2	7
85 and over	15	34	21	78
**Level of physical limitations ^2^**				
Mild	10	23	1	4
Moderate	16	36	4	15
High	9	20	7	26
Very high	9	20	15	56
**Mobility**				
Only in the home	15	34	15	56
Also outside the home with help ^3^	29	66	12	44
**Monthly income brackets (EUR)**				
Up to 600	5	11	1	4
601–1500	32	73	17	63
1501–2500	6	14	7	26
2500+	-	-	2	7
Missing	1	2	-	-
**Total respondents with DHH-PCA ^4^**	44	100	27	100

^1^ Three cases with both DHH and PCA (respondents with high/very high limitations, and episodes of falls in the home); ^2^ The level of physical/functional limitations is based on 12 ADLs-IADLs, two mobility limitations (going up/down the stairs and bending to pick up an object), plus sensory limitations in hearing and seeing. Mild = no activities “not able”, Moderate = one–two, High = three–four, Very high = five or more; ^3^ Respondent is able to leave the house, at least two times a week, only if accompanied or with aids (cane, walker); ^4^ One male DHH and two males PCAs.

## Data Availability

The quantitative data supporting the findings of the study are not publicly available due to privacy/ethical restrictions. There is indeed confidential information that could compromise the anonymity of research participants as potential indirect identifiers, e.g., city of residence. Moreover, the dataset includes: small denominators (the number of participants is low = 27, less than 100); and small numerators (several values count less than three units). Also, original verbatim transcriptions in the charts are not publicly available due to privacy/ethical restrictions, that is to their containing information that could compromise the privacy/anonymity of research participants. (e.g., include names of persons and locations and other potential identifiers of respondents). However, a dataset regarding the sample of the main study carried out in 2019, is openly available in Mendeley at https://doi.org/10.17632/3ryrpz224h.2 (accessed on 15 February 2022).
